# Conformational Changes and Slow Dynamics through Microsecond Polarized Atomistic Molecular Simulation of an Integral Kv1.2 Ion Channel

**DOI:** 10.1371/journal.pcbi.1000289

**Published:** 2009-02-20

**Authors:** Pär Bjelkmar, Perttu S. Niemelä, Ilpo Vattulainen, Erik Lindahl

**Affiliations:** 1 Center for Biomembrane Research & Stockholm Bioinformatics Center, Department of Biochemistry & Biophysics, Stockholm University, Stockholm, Sweden; 2 VTT Technical Research Center of Finland, Espoo, Finland; 3 Department of Applied Physics, Helsinki University of Technology, Helsinki, Finland; 4 Memphys—Center for Biomembrane Physics, Physics Department, University of Southern Denmark, Odense, Denmark; 5 Department of Physics, Tampere University of Technology, Tampere, Finland; Weill Medical College of Cornell University, United States of America

## Abstract

Structure and dynamics of voltage-gated ion channels, in particular the motion of the S4 helix, is a highly interesting and hotly debated topic in current membrane protein research. It has critical implications for insertion and stabilization of membrane proteins as well as for finding how transitions occur in membrane proteins—not to mention numerous applications in drug design. Here, we present a full 1 µs atomic-detail molecular dynamics simulation of an integral Kv1.2 ion channel, comprising 120,000 atoms. By applying 0.052 V/nm of hyperpolarization, we observe structural rearrangements, including up to 120° rotation of the S4 segment, changes in hydrogen-bonding patterns, but only low amounts of translation. A smaller rotation (∼35°) of the extracellular end of all S4 segments is present also in a reference 0.5 µs simulation without applied field, which indicates that the crystal structure might be slightly different from the natural state of the voltage sensor. The conformation change upon hyperpolarization is closely coupled to an increase in 3_10_ helix contents in S4, starting from the intracellular side. This could support a model for transition from the crystal structure where the hyperpolarization destabilizes S4–lipid hydrogen bonds, which leads to the helix rotating to keep the arginine side chains away from the hydrophobic phase, and the driving force for final relaxation by downward translation is partly entropic, which would explain the slow process. The coordinates of the transmembrane part of the simulated channel actually stay closer to the recently determined higher-resolution Kv1.2 chimera channel than the starting structure for the entire second half of the simulation (0.5–1 µs). Together with lipids binding in matching positions and significant thinning of the membrane also observed in experiments, this provides additional support for the predictive power of microsecond-scale membrane protein simulations.

## Author Summary

Proteins that transport ions across the cellular membrane are essential for cellular life. The proteins conducting positively charged potassium ions are key players in heart beat and nerve impulse generation because they are regulating the electrical excitability of the cell (together with proteins transporting other ions). These particular ion channels open and close in response to voltage changes across cellular membranes, but the details of this process are still not fully understood. It is, however, known that the main protein element responsible is a helical section containing several charges. Through new computer simulation methods, we have been able to run unprecedentedly long atomic simulations of an entire potassium channel embedded within a patch of membrane to help to shed new light on this gating process. Upon changing the voltage across the membrane, we observe a change in structure of this helical protein segment that appears to be an early sign of transition from the open to the closed state of the channel. This has also been previously proposed to be critical for the gating process. Understanding these structural changes on an atomic level is essential for both advancing basic science and enabling drug design targeting of voltage-regulated ion channels.

## Introduction

Potassium channels are the single most common type of ion channels in nature. The subclass of voltage-dependent potassium channels enable controlled ion transport over the cell membrane and are hence pivotal for a wide range of functions such as nerve impulse action potentials, our heart beats, insulin secretion upon low ATP, and many diseases [1]. The first X-ray structure to be determined was the bacterial tetrameric KcsA K^+^ channel [2], which is pH-modulated with an opening mechanism likely controlled by protonation and salt bridges/hydrogen bonds [3]. Recently determined structures of voltage-modulated potassium channels such as KvAP [4],[5] and Kv1.2 [6],[7] in the open state share the same central pore domain (PD), but for these the actual voltage-sensitivity is introduced by adjacent 4-helix voltage sensor domains (VSDs) in each monomer. The PD is formed by eight transmembrane helices (and their connecting loops), two from each subunit, and it contains an ion-conducting channel connecting the inside and the outside of the cell. This water-filled pore consists of a rather large intracellular cavity leading into a narrow ion-selectivity filter at the extracellular end.

The access to the intracellular cavity, and hence to the ion-conducting pore, is controlled by a gate that is opened in response to the membrane potential. It is well established that the gating within the PD is regulated by conformational changes in the VSDs in response to the varying membrane potential. In particular, the gating is caused by the voltage sensor S4 helix of the VSD which contains several positively charged amino acids [8]–[10]. When the charged S4 helix moves it creates an experimentally measurable current called the gating current, which is distinct from the so called *α*-current caused by ions passing over the membrane through the protein channel. The actual opening of the channel pore domain and initiation of the *α*-current has experimentally been shown to occur in the millisecond range [11], but Sigg et al. have reported an early component of the gating current with a time constant as short as 12 µs [12].

**Figure 1:**
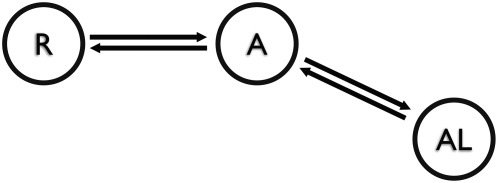
Model of voltage gating. Based on Villalba-Galea et al. [27], not including the RL state. At hyperpolarization the VSD is in resting (R) state and protein is not conducting. Upon depolarization it transfers to an activated (A) state, likely due to S4 voltage-dependent motion and coupled to the channel opening. This state is transient and converts into a more stable relaxed state (AL) at prolonged depolarization (non-conducting). Crystal structures likely correspond to this open-inactivated state.

When the cell is at rest, the membrane is hyperpolarized (negative potential on intracellular side) and the gates of the voltage-sensitive potassium channels are closed, their S4 helices located near the intracellular membrane border, referred to as the down or “resting” (R) state in Figure 1. Upon depolarization of the membrane, channel opening is thought to occur in a stepwise fashion where each voltage-sensing domain first activates independently, by transferring some of the positive charges within the voltage sensor helix from the intracellular side in the down state to the extracellular side of the membrane, the up state. In this transient activated state of the protein, the ion-conducting pathway is still obstructed by the gate formed by the intracellular ends of the four pore-lining S6 helices of the PD. Subsequently, the channel gate is opened by an additional cooperative motion [13], yielding the open-activated state (A) in Figure 1. For long periods of depolarization, the channel is believed to undergo slow inactivation caused by conformational changes around the selectivity filter [14]–[17], making it impermeable for ions even though the gate is open (the open-inactivated state, AL). Going from R over A to AL, i.e., opening and eventually inactivating the protein after long depolarization, is referred to the forward direction in literature. However, since the experimental structure corresponds to the AL (or possibly A) state, the current simulations aim to assess the conformational changes in the backward direction, both due to hyperpolarization and equilibration in a more fluid bilayer.

Several experimental studies have shown that the gating involves displacement of just over three charges per subunit across the membrane [9],[10],[18],[19]. Although the exact nature of the gating process is not yet fully understood, several ideas have been proposed, for instance S4 rotation combined with crevice reshaping to effectively move charges between inside/outside (transporter model [20]), larger rotation and translation of S4 inside a rigid environment (helical screw [21],[22]) or S3b/S4 helices moving as a hairpin (paddle model [4]). These models have mostly converged at smaller necessary S4 displacements combined with rotation/tilt, but there are still important differences, for instance whether S4 moves independently of S3b or not, whether the charged arginine residues at some point are facing the membrane or not, and in which order the motion events occur [23]. Note that small S4 displacements still could explain the effective charge displacement due to significant water depressions in the VSDs that cause local focusing of the electric field [24]–[26].

Additional interesting conformational changes upon gating have recently been proposed. In the new Kv1.2 crystal structure [7], the intracellular end of the S4 helix adopts a 3_10_ conformation leading the authors to propose that this secondary structure alteration is relevant for the gating. This is also supported by recent data from Villalba-Galea et al. [27] suggesting that the R to A transition of the voltage sensor takes place as 3_10_ helix whereas the A to AL transition changes the voltage sensor conformation to *α*-helix.

The experimental structures in combination with theoretical models of the closed state [28] has led to considerable interest in understanding these structural transitions. Simulations of isolated voltage sensors have confirmed water-filled VSD crevices (on both intracellular and extracellular sides), S4 stabilization and field focusing [29],[30]. Treptow et al. used short (9 ns) simulations of an integral Kv1.2 channel to show how the S4 gating charges can be stabilized [31], and Jogini/Roux have reported on structural flexibility and arginine side chain dynamics in the voltage sensors from 20 ns-simulations [26],[32]. Coarse-grained representations of Kv1.2 systems [33] have been used to reach 350 ns of simulation, although the pore in this case collapsed to an apparently closed state even without any applied field. Recently, Nishizawa et al. reported on S4 motion in an isolated VSD when running at elevated temperatures applying strong fields of 0.15 V/nm and position-restraining adjacent helices. While interesting, it is unclear how realistic this is since the position restraints will prevent side chains in adjacent helices from stabilizing S4, and near-instantaneous membrane rupture was observed at 0.2 V/nm (in our longer simulations, fields exceeding 0.1 V/nm lead to electrostatic breakdown in 100 ns) and no significant S4 motion occurred in 30–40 ns simulations at lower voltages [34]. While all these studies provide new insights into Kv1.2 dynamics, they are all partly limited, either by short timescales or other approximations; VSDs might for instance behave differently than integral ion channels, and any charged elements will move if the voltage applied is high enough and the rest of the structure is restrained. Ideally, one would want to simulate an entire ion channel system for long times at low field strengths, which previously has not been possible.

Here, we present a full atomic detail microsecond simulation of an integral Kv1.2 system comprising 120,000 atoms, with an applied field of 0.052 V/nm, corresponding to approximately 500 mV of potential drop over the membrane (see methods and Figure S1). The seed for this project was our recent development of new parallelization techniques in the GROMACS molecular simulation code [35] that have enabled us to reach a microsecond with high-accuracy simulation settings in just a month of runs (roughly 125,000 CPU-hours). Analysis of the S4 helix structure, dynamics and interactions in applied fields is of course of particular interest. Another challenge is to compare and contrast results to the new high-resolution chimera Kv1.2/Kv2.1 structure [7] that appeared during the project, and where the intracellular part of S4 assumes 3_10_ helix conformation. The primary questions we wanted to address are whether previously observed motions in short ion channel and VSD simulations do represent the early stages of transition towards the resting state or merely short-time fluctuations? To what extent can molecular simulations started from lower-resolution structures predict native conformations and/or lipid-protein interactions, and can we use it to better understand channel opening/closing?

## Methods

### System Assembly

**Figure 2:**
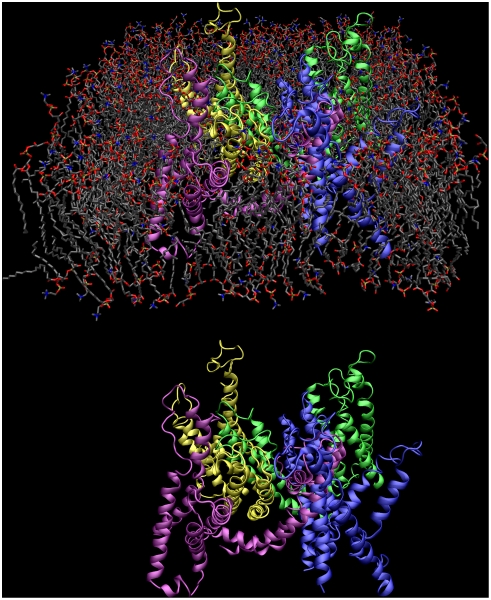
Kv1.2 immersed in a membrane. The protein is colored by subunits (blue, yellow, purple, green) and lipids chains are drawn in gray. Water molecules were left out for clarity. The full system consists of roughly 120,000 atoms. The lower panel shows the same system without lipids.

The initial ion channel conformation used in this study was constructed from the X-ray structure of the Kv1.2 channel [6], PDB accession ID 2A79. The monomer coordinates were assembled into a tetramer channel which kindly was provided directly from MacKinnon's laboratory. To reduce the system size the T1 domain was excluded. The parts used were chains B, from residue T219 (inclusive), C from UNK33 corresponding to A162 in Kv1.2, and chain D. Since parts of the transmembrane helices were of limited X-ray resolution it was necessary to complement it with side chain and loop modeling to arrive at a model suitable for simulations. The voltage sensor domains together with the pore domain helices from the separate chains were structurally aligned to the corresponding part in the ROSETTA model of the open state of the Kv1.2 channel [28], using the program STRUCTAL [36]. Subsequently, the missing parts of the crystal structure (i.e., residues denoted UNK, missing side chains and loops) were copied from the ROSETTA model structure. This essentially resulted in a model using the experimental data for the backbone and most side chains, with the remaining side chains and missing loops from the ROSETTA model. The ion channel structure was immersed in a lipid membrane by using the Membrane package of VMD [37]. Starting from a pure POPC bilayer, the head group of every fourth lipid was randomly (in each monolayer) exchanged for the negatively charged PG head group. The ion channel was subsequently inserted and overlapping lipids deleted, after which 313 POPC and 111 POPG lipids remained in the membrane. Although somewhat similar to a prokaryotic membrane, this mixture was used to better mimic conditions used in the crystallization protocols [6],[7] and it has previously also been used in VSD simulations [38]. The negative head groups in particular could have important interactions with arginine, and PG force field parameters are better tested than PS. The bilayer was first melted with MD at 300 K, while the protein was kept frozen and the z-coordinates of the lipid chain methyl groups position restrained (*F_c_* = 1000 kJ/mol/nm^2^) to keep the membrane intact while it packed around the protein for 1 ns. The system was subsequently solvated with 26,632 SPC waters [39], of which 127 were replaced with potassium ions to neutralize the net system charge, followed by another 1 ns of equilibration with only the protein frozen. The final assembly reached 119,913 atoms, and an approximate system size of 12*12*10 nm^3^ (Figure 2).

### Molecular Simulations

The protein part of the system was described with the OPLS-AA/L force field [40], and the lipids with the Berger force-field [41]. The reason for this choice is simply that the Berger force field has been shown to accurately reproduce experimental results [42],[43] combined with a low computation cost due to the united CH_2_ atoms in the lipid chains, and then it is natural to combine it with OPLS from which the Berger FF was derived. The OPLS-AA combination rules and 1,4 scaling factors (0.5) were used when mixing united and all-atom models. Atomic names of the VMD lipids were converted with an in-house script (available upon request).

All simulations were performed with a development version of GROMACS 4.0 [35],[44] that enabled efficient scaling and high performance (50–60 ns/day) using 170 cores on a Cray XT4 supercomputer. Bond lengths were constrained with the LINCS algorithm [45] while SETTLE [46] was used for water molecules. A newly developed non-iterative parallel constraints algorithm (P-LINCS) [47] enabled us to introduce virtual interaction sites to remove all internal vibrational degrees of freedom of hydrogens even when using domain decomposition, which in turn made it possible to extend time steps to 5 fs while maintaining energy conservation [35]. (All software is freely available through http://www.gromacs.org.) Electrostatic interactions were calculated every step with the Particle-Mesh Ewald algorithm [48]. Due to the slightly worse scaling properties of PME (3D Fourier transforms) it proved efficient to move interactions to direct space by using longer cutoffs of 12 Å both for PME and van der Waals interactions, so the PME grid cell dimensions could be reduced to 88×88×68 (for technical details, see Ref. [35]). Neighbor lists were saved and reused for 5 steps. All simulations were performed at constant temperature and with semi-isotropic pressure scaling. The temperature of the system was coupled to 300 K using the weak coupling algorithm with a time constant of *τ_T_* = 0.1 ps [49]. The X+Y (isotropic) and Z box dimensions were coupled independently to reference pressures of 1 bar with Berendsen weak coupling, a *τ_P_* = 10.0 ps time constant, dispersion corrections to pressure, and a system compressibility of 4.5·10^−5^ bar^−1^ [49].

The assembled system was equilibrated in three steps with gradually weaker position restraints on the protein (1000 kJ/mol/nm^2^ for 1 ns, 100 kJ/mol/nm^2^ for 10 ns, 10 kJ/mol/nm^2^ for another 10 ns). Side chains were only restrained in the first of these runs. Production runs were performed without restraints; an initial 50 ns without applied potential followed by a microsecond simulation with an applied electrical field of 0.052 V/nm along the z system axis, with lower potential on the intracellular side. With a box length of ca 10 nm in the z direction, this corresponds to a potential drop of ∼500 mV, which is due to depolarization at water/lipid interface and will occur mostly over the membrane part (Figure S1). The treatment of external potentials in membrane systems has recently been covered in detail [50], and it has also been reported that the periodicity effects of the PME algorithm artificially can increase polarization, corresponding to almost 50% higher field in pure water [51].

As additional tests, the non-polarized simulation was extended to 0.5 µs, and to assess the effect of higher temperature/potential a separate third 0.5 µs simulation was started from the conformation after 0.8 µs of lower applied field, but increasing temperature to 343 K and field strength to 0.1 V/nm. Finally, a 50 ns test simulation (starting from 1 µs) was performed where the interactions between R300-E183 and R303-E226 were turned off using energy exclusion groups in GROMACS.

## Results

### Microsecond-Scale Dynamics of the Kv1.2 Channel

In terms of structural stability there are two obvious factors that could affect the integrity of the ion channel negatively in extended simulations; the significant number of rebuilt side chains and the complete removal of the non-membrane-spanning T1 domain believed to be partly responsible for tetramerization/stabilization of the Kv1.2 chains [6]. Microsecond scales are still quite hard to reach even for small globular proteins without the complex long-range electrostatics critical in membrane and ion channel systems, and to our best knowledge it has not previously been performed for systems of this size. This is particularly critical since the slow lipid diffusion and reorientation slows down all motion in the membrane, so full stabilization (or unfolding) of a structure should be expected to take at least hundreds of nanoseconds for a membrane protein.

We directly observe distortion of the membrane close to the protein with significant local bending, lipids pointing in toward polar groups and deep water depressions (though not full transmembrane pores) forming in all voltage sensor domains, just as previously observed in shorter simulations both of isolated voltage sensor domains [29],[38] and complete ion channels [26],[31],[52].

**Figure 3:**
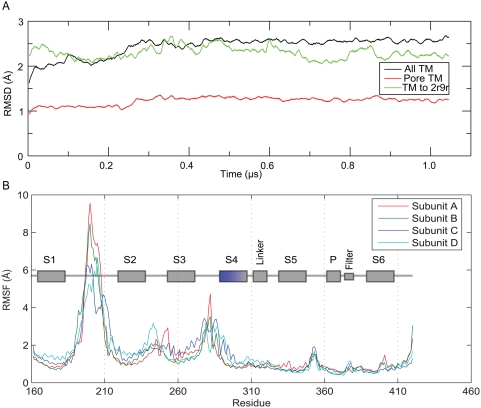
Protein flexibility. (A) Root-mean square displacement (RMSD), smoothed by a 1 ns running average, of the transmembrane Cα atoms in the entire protein (black), and for the pore domain (red), compared to initial simulation conformation and to the new chimera crystal structure of Kv1.2 (PDB ID 2R9R) (green). (B) Root-mean square fluctuation (RMSF) of Cα, with each subunit displayed separately. The secondary structure regions of the protein are identified for clarity.

The C*_α_* coordinate root mean square displacement (RMSD) of the transmembrane helices relative to the (2.9 Å resolution) crystal structure rapidly increases during the initial 50 ns of free equilibration, but levels off around 2 Å. When the hyperpolarization field is applied it very slowly (0.25 µs) continues to grow to 2.6 Å, where it is stable for the remainder of the simulation (Figure 3A). The central pore-lining helices are even more rigid, although they are actually part of four different chains. For the first 0.2 µs they are roughly within 1 Å of the crystal structure, after which it increases just slightly to 1.2 Å as a consequence of the other structural changes in the rest of the structure. Since this was achieved *without* the tetramerization domain attached this suggests the extra domain might actually not be critical to maintain the Kv1.2 structure, at least on microsecond scales. The structural flexibility of the voltage sensors has previously been reported by Jogini [26] and is quite striking; one possible explanation is that it could be necessary to enable the structural transitions required for the channel function.

Since the starting model was derived from the Kv1.2 crystal structure [6] in combination with the ROSETTA open model [28] it is quite illustrating to compare the RMSD of corresponding parts of the transmembrane (TM) helices to the newer 2.4 Å chimera structure, PDB accession ID 2R9R [7]. As the simulation proceeds beyond 0.3 µs the RMSD of the transmembrane part shows that the protein becomes less similar to the starting model, leveling out at about 2.6 Å, compared to the new crystal structure to which the RMSD is 2.2–2.3 Å. Thus, even when starting from imperfect coordinates, the simulation ensemble is closer to the new high-resolution chimera structure. However, as recently addressed [27],[53], it is not trivial to interpret the crystal data: the current structures of Kv1.2 might not have captured the protein in its open-activated state but rather in the open-inactivated state due to prolonged depolarization.

To separate inherent flexibility from simple drift/unfolding of the different Kv1.2 domains the C*_α_* root mean square fluctuations (RMSF) around the average structure of each chain from the 1 µs-simulation were calculated (Figure 3B). All four subunits exhibit virtually identical patterns, with the two largest peaks in the curve correspond to the large flexible extracellular loops. Experiments have shown that these two loops can be omitted and the resulting protein remains functional [7]. At least the endpoints of the S3&S4 helices are more mobile than the S1&S2 helices, and the pore helices are quite rigid. Interestingly, the protein regions located “behind” the selectivity filter in the three-dimensional structure (the C-terminal part of the S5 helix and the P-loop in residues 361–373, together with the selectivity filter itself in residues 374–378) proved remarkably stable throughout the simulation. This is consistent with experimental data; residues in the P-loop in the highly homologous pore domain of the KcsA potassium channel have been shown to be involved in filter-stabilizing interactions [17]. Two potassium ions were located within the selectivity filter throughout the simulations and no events of ion transfer through the filter were recorded.

While stable, the ion channel is still quite a sensitive system, which became obvious for the high-temperature/field (343 K&0.1 V/nm) simulation. The membrane stayed more or less intact over 500 ns, but the RMSD of the TM part gradually increased to 5 Å (*data not shown*). This was coupled with very large distortions of the VSDs and even partial unfolding of alpha helices. In our opinion, the system is simply unfolding under these conditions. Fields higher than 0.1 V/nm consistently lead to electrostatic breakdown on scales of 100 ns in test simulations. As recently reported by Böckmann et al. there appears to be an exponential dependence of pore formation time vs. applied field [51], and we therefore decided to limit the analysis to the simulations closer to long-time stable conditions.

Perhaps surprisingly, we only observed a couple of Ångström of S4 helix translation along the bilayer normal towards the intracellular side and no effect on the pore radius was observed. Since the transition between open and closed conformations of the channel is a process of one or a few milliseconds [11], it is not expected to occur completely in µs-scale simulation. There is however a small but significant component of the gating movement, the fast gating component, which appears to occur on µs timescales [12] and it is consequently feasible that initial stages of channel structural transitions could be captured by the current simulation. The fact that the crystal structure might not be in the open-activated state (A) but rather the open-inactivated state (AL) is also important since even when hyperpolarization is applied the structure might have to go through the A state before being able to access resting conformations, a process not expected to be coupled to any significant charge transfer [27].

### S4 Motion and Stabilization

To characterize the behavior of the key charged residues in the S4 helix, the movement along the z-axis (parallel to the membrane normal) was tracked. This is non-trivial since the protein position fluctuates a lot over a microsecond due to membrane undulations. It was thus calculated as the difference in the z-coordinate of the C*_α_* atoms of these residues after removing the rigid body motion of the non-S4 helices of each VSD by fitting each frame of the simulation trajectory to the corresponding C*_α_* atom of the starting structure of the protein. The hydrogen-bonding pattern of these cationic side chains were also analyzed since conformational changes often are associated with alterations in the hydrogen-bonding.

**Figure 4:**
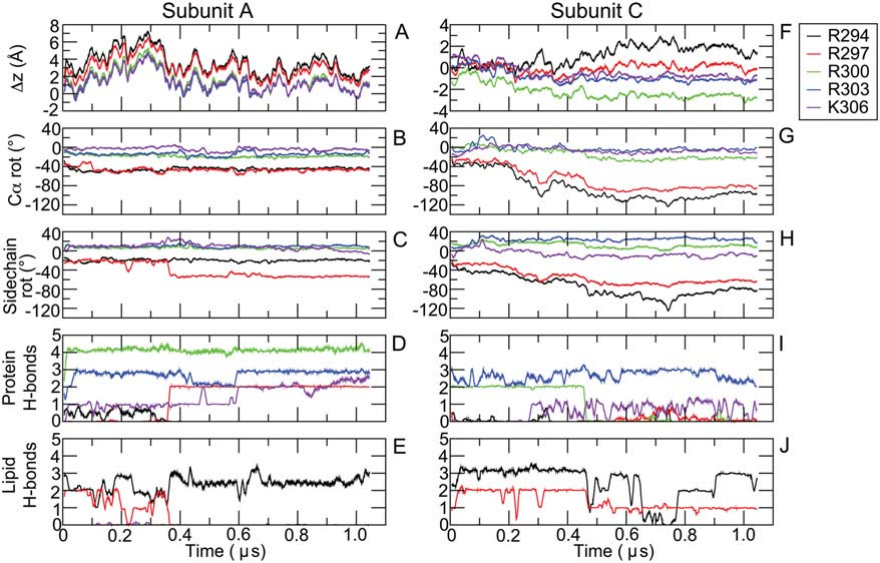
Dynamics of charged amino acids in the S4 helix of subunits A and C. Panels A & F show the relative Cα translation along the membrane normal, with positive direction towards the extracellular side. Note the difference in scales on the y-axis. Panels B/G & C/H indicate rotation of Cα and the outermost heavy atom around the local helix axis, respectively (clockwise rotation being positive when viewed from the N-terminal). Panels D/I & E/J display the number of hydrogen bonds formed with the rest of the protein and lipids, respectively (acceptor-donor distance<3.5 Å, angle<30 degrees). A 1 ns running average is used in all panels.

The relative displacement of the C*_α_* atoms along the z-axis (after removal of overall VSD movement) is shown in Figures 4 (A & C subunits), S2 (B & D subunits), and S3 (A & C subunits, depolarized simulation).

**Figure 5:**
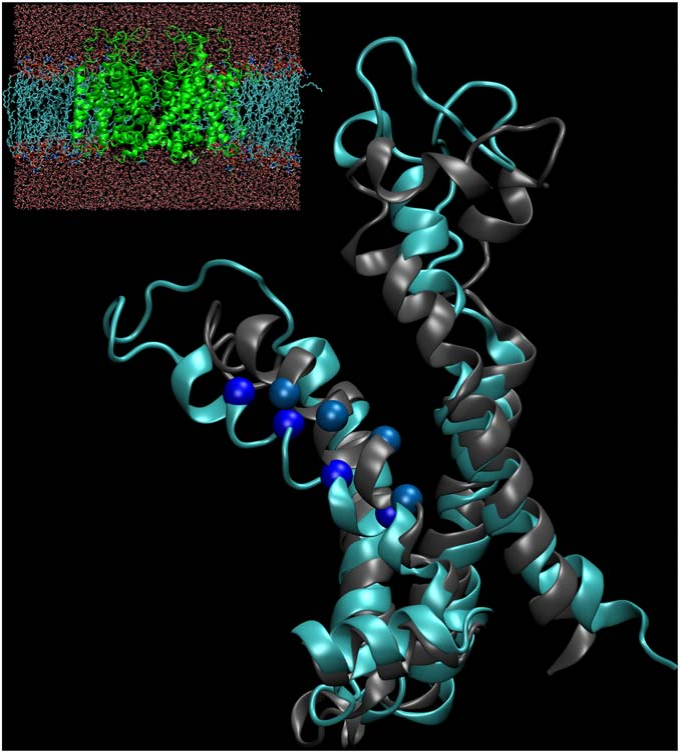
Sideview of subunit C, helix S1 to S4–S5 linker helix. For a clearer view of the S4 helix, the protein is visualized as viewed from the pore domain. The extracellular side is “up” in the figure. The starting structure (gray) and the last frame of the simulation (cyan) are shown. They were structurally aligned using the Cα coordinates of the helices S1 to S3. In both structures, the Cα atoms of the four top arginine residues (R294, R297, R300 and R303) in S4 are shown as blue spheres for comparison. Simulation box is visualized in inset (same orientation).

In general, fluctuations of 5–10 Å were seen over hundreds of nanoseconds, suggesting that it is quite difficult or impossible to draw conclusions from shorter simulations. However, it is quite striking that there is really only one case—subunit C—where we see significant changes due to the applied field (for reference: simulations had identical starting conditions). The net movement towards the intracellular side is quite low in all cases (with and without applied field), up to ∼3 Å for R300 in subunit C, which really is the same order of magnitude as the fluctuations on microsecond scale. R303 and K306 also moved marginally in the same direction while R294 adopted a more extracellular position hence extending the N-terminal part of the helix. A visually more appealing way to represent this is depicted in Figure 5. It shows that the S4 helix in this subunit is less bent due to straightening of the extracellular portion of the helix (see next section). It has the effect that the mass center of the S4 helix backbone is shifted towards the intracellular side, especially the N-terminal half, explaining the relative downward movement of R300 but also the lack of corresponding movement in R294 and R297 which both end up on a more extracellular phase of the helix (due to rotation) compared to the reference structure.

Hydrogen-bonding patterns of the key cationic *side chains* are also displayed in Figures 4, S2, and S3. Hydrogen bonds formed with other protein residues and lipids are shown, not with water, but in practice all hydrogen bonding donors and acceptors in these residues are matched. Consequently, the total number of hydrogen bonds formed by an arginine is 5 (3 for lysine), one for each side chain hydrogen connected to an electronegative atom (nitrogen). R294 and R297 formed strong hydrogen bonds with oxygens in the lipid head groups in the extracellular leaflet and water molecules in the extracellular VSD crevice but they also interacted transiently with residues in the neighboring S5 helix of the pore domain (D352 and E353). R300 and R303 on the other hand, mostly formed stable salt-bridges with negatively charged residues within the VSD, in particular E183 in S1 and E226 in S2, something that also has been shown in the crystal structures of Kv1.2 and in other modeling/simulation attempts of the open *Shaker* channel [26],[28],[54], and with water (in the extracellular crevice). For subunit C, where we did see clear differences with/without hyperpolarization, the formation of new hydrogen bonds with the protein and water in the extracellular VSD crevice instead of interaction with lipids (for example in R297 in subunit A and similar events in the other subunits, B and D) was highly correlated with a large rotation around the helical axis (see next section). As the rotation in the extracellular half of the S4 helix took place in subunit C under hyperpolarization, the total number of hydrogen bonds formed between lipids and R294 and R297 decreased as these side chains gradually reduced their exposure to the lipid environment. The lysine residue, K306, formed hydrogen bonds with protein, first with E236 in S2 (except in subunit C) and later with D259 in S3 (interactions confirmed by experimental findings in Shaker [55]), and water molecules in the intracellular VSD crevice. Interestingly, the formation of the hydrogen bond between K306 and D259 in subunit C at 0.3 µs is highly correlated to onset of 3_10_ helix growth in S4 of subunit C.

The charge movement in S4 towards the intracellular side upon channel open-to-closed gating is thought to be associated with hydrogen-bonding pattern changes. The two most extracellular arginine residues should, as they move down in response to the hyperpolarization, start competing for the hydrogen bonds formed between the following two arginine residues (R300 and R303) and the anionic residues in the VSD center (E183 and E226). As they do, these latter two arginine residues become accessible to the water in the intracellular crevice and/or other polar/charged residues residing there [56]. It is likely that these changes in hydrogen-bonding within the VSD contribute to the free energy barrier between the intermediate states of gating. Curiously, no systematic change in this hydrogen-bonding pattern was observed, with the exception of R300 in subunit C under hyperpolarization, where we see a complete loss of its hydrogen bonds to protein.

This could of course be due to the fact that this process is much slower compared to the simulation timescale, as might be possible if this really is the main free energy barrier of structural transformations between the open and closed states. To investigate this further we attempted to selectively exclude the interactions between the residues participating in these hydrogen bonds in the open state of the channel. After 50 ns of such a simulation (continuing from the last frame of the normal hyperpolarization simulation at 1 µs) we still did not see any of these structural and binding rearrangements. This indicates that the free energy barrier could rather be attributed to the hydrophobic area surrounding S4 in the central part of the VSD, formed by isoleucine residues in S1 and S2 (corresponding to the Kv1.2 residues I177 and I230 respectively) as proposed by Campos [57] and/or by phenylalanine (F233) as proposed by Long [7], separating the extracellular and intracellular water crevices. To fully close the channel, the S4 helix must be translated towards the intracellular side according to all current models. It will force its arginine residues to cross this hydrophobic area devoid of possible hydrogen-bond acceptors, and hence the hydrogen bonds between the arginine residues and the anionic residues located extracellularly of the hydrophobic area (E183 and E226 in particular) are almost certain to break as the former move across the barrier without stabilizing hydrogen bonds, which would lead to a kinetic free energy barrier for this transition. Once the side chains have crossed the hydrophobic area, they can again form hydrogen bonds with other polar residues of the protein or with water in the intracellular crevice of the VSD.

### Secondary Structure Alteration Might Be Necessary for S4 Translation

Rotation of the S4 helices was observed and quantified by measuring the local rotation for all residues within the helix in each subunit with respect to the position of the corresponding residue in the starting conformation of the protein, defined from a local helical axis. For the key positively charged residues in S4—the four most extracellular arginine residues (R294, R297, R300 and R303) and the downstream lysine residue (K306)—both the rotation of C*_α_* and the outermost heavy atoms were analyzed (Figure 4 for subunits A & C, Figure S2 for subunits B & D).

**Figure 6:**
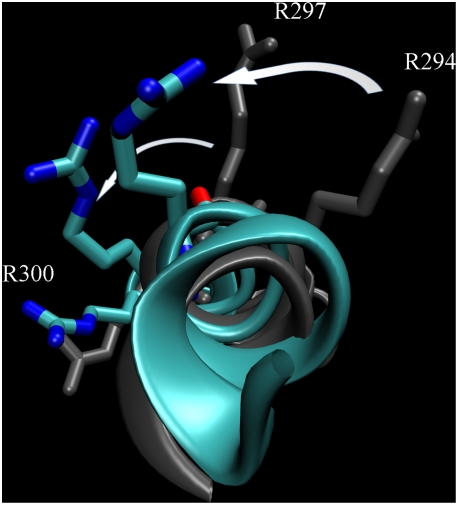
Rotation of the S4 helix in subunit C. An extracellular view of the S4 helix at the beginning (gray) and end (cyan) of the simulation. The top three arginines in each conformation are shown in stick representation and the large counter-clockwise rotations of R294 and R297 indicated by arrows. The end-conformation was been aligned to the initial using the Cα atoms in helices S1 to S3 after which the S4 helices were aligned to each other in order to compare their relative rotation.

Interestingly, S4 helices in all subunits exhibit a limited rotation in *all* simulations, even in the depolarized state. Significant rotations were always counterclockwise around S4, as seen from the extracellular side, turning the two most N-terminal arginine residues slightly toward the VSD interior (see Figure 6). All four subunits had a similar initial behavior with respect to S4 rotation (Figures 4 and S2, S3 in depolarized state); a rotation of around −30 to −40° for the C*_α_* atoms of R294 and R297 while their side chains underwent a somewhat smaller rotation at the beginning due to favorable hydrogen bonds to lipid head groups. Some of these side chains were subject to additional rotations, highly correlated with changes in their hydrogen-bonding, as is apparent for R297 in subunit A at ∼370 ns (and similar events for R294 and R297 side chains in subunits B and D, Figure S2), where the creation of two hydrogen bonds with protein caused the side chain to rotate to the level of the C*_α_* atom. The more intracellularly located residues in the helix, e.g., R300/R303/K306, underwent marginal rotation, if any.

In addition to the small but clear rotation of the S4 helix present in all chains, the extracellular half of the helix in subunit C also exhibited a significantly larger but slow rotation in the 0.3–0.7 µs timeframe when the hyperpolarization field was applied. R294 and R297 rotated about −80 to −120° (peak values as much as −150°), whereas the minimal rotation of R300 and R303 was kept. This had the consequence that the helix became longer as the pitch angle between helix turns increased and the positively charged residues in the helix aligned on the same phase, all pointing toward the VSD interior, forming a significant stretch of 3_10_ helix. The C*_α_* rotation is again closely correlated with side chains (the backbone might even precede the sidechains). Together with the rearrangement taking 0.4 µs to complete, it indicates a predominantly entropy-limited process.

To double-check that the *α* helix vs. 3_10_ helix states are correctly modeled, the energies for an ACE-Ala_20_-NME peptide in the two secondary structures were calculated, confirming that the *α* helix enthalpy is lower by roughly 5 kJ/mol per residue. This agrees well with earlier studies using both OPLS [58] and other parameters [59] that also calculated an entropic contribution to oppose roughly 1/3 of this. The final free energy is thus roughly 3–4 kJ/mol lower per Alanine residue for an alpha helix in solvent, with a low barrier around 1 kJ/mol (from 3_10_ to *α* helix) in earlier OPLS studies [58]. In addition, the force field employed here has also been succesful at reproducing the free energy landscape for the 3_10_ helix-containing Trp-cage protein to within 1.5 Å [60], so we do not expect the transition to be an artifact from parameters.

The larger rotation was a progressive event, not associated with single hydrogen-bonding formation/breakage as in the abrupt R297 side chain rotation in subunit A. Note that no corresponding rotation occurred in any of the subunits in the simulation without external field. The 3_10_ helix extension (which even starts from the intracellular side) was associated with formation of a new hydrogen bond between K306 and D259. S3b motion was clearly coupled with S4 in fluctuation motions and initially even rotates slightly counter-clockwise around S4 to maintain interaction surfaces. However, at least in the present simulations, these interactions were broken when S4 rotated >70° degrees, and the extracellular part of S4 turned almost a third of a turn relative to S3b. Hydrogen-bonding to lipids decreased in R294 and R297 after approximately 470 ns (in favor of hydrogen bonds to water and to some extent protein residues in the case of R297), but most of the rotation had already taken place at that point. Consequently, the lipids involved in hydrogen-bonding with these two arginine residues were dragged in towards the VSD/PD interface as the field-driven subunit C rotation took place.

**Figure 7:**
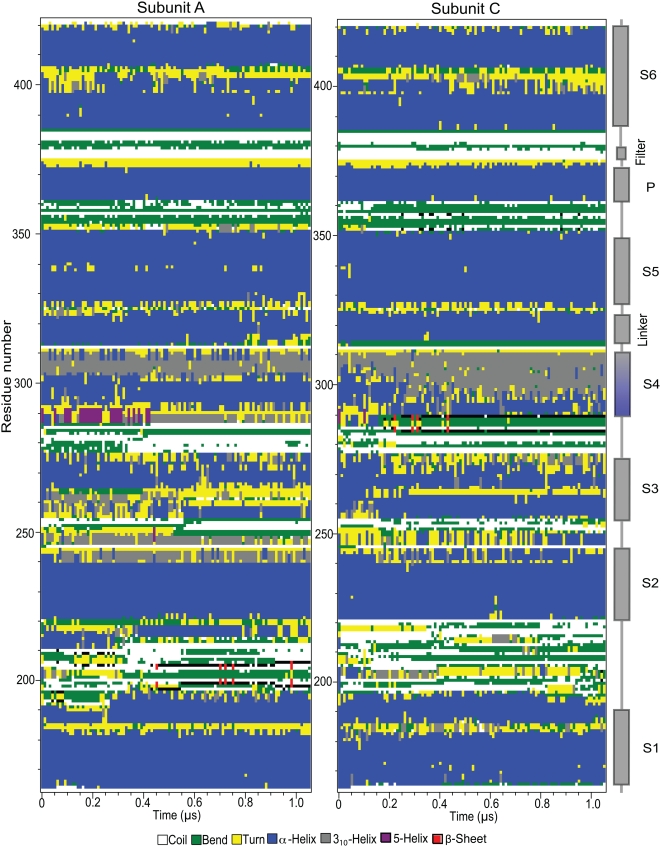
The secondary structure of subunits A and C as calculated by DSSP. Note the significant growth of the 3_10_ helix in subunit C that correlates with S4 rotation.

Secondary structure plots from the hyperpolarization simulation are presented in Figures 7 (subunits A,C) and S4 (subunits B,D), while Figure S5 shows the secondary structure in subunits A,C in the non-polarized simulation. They provide additional evidence of protein stability; the S1–S6 transmembrane helices keep their core *α*-helical structure while the helix ends and the connecting loops are somewhat more variable in their secondary structure as expected. The region around the selectivity filter (residues 274–278) also shows a high structural stability throughout the simulation.

The rotation of the extracellular half of the S4 helix in subunit C caused this part of the helix to be more tightly wound and somewhat elongated because the pitch angle between following helix turns increased. The secondary structure in this region accordingly underwent a change from *α*-helix to 3_10_-helix. In fact, the handful most C-terminal residues of the S4 helix in all subunits adopt 3_10_ secondary structure already early in the simulation. However, the significant 3_10_-helix growth in S4 of subunit C starts after some 200 ns and continued for 250–300 ns, perfectly correlated with the rotation of the extracellular end of this helix. After the rotation, the 3_10_-helix consistently encompassed a full 17 residues in S4 (residues 293–309), including the two most extracellular arginine residues R294 and R297. No corresponding 3_10_-helix growth was seen in any of the subunits in the simulation without the applied electric field.

Our model was derived from the original crystal structure of the Kv1.2 channel [6] but it has very interesting similarities to the new fully-resolved Kv1.2 chimera crystal structure [7]. In particular, the new structure features 3_10_-helices C-terminally of the second arginine in S4 (R297). One idea put forward by MacKinnon and coworkers is that the 3_10_ portion of the secondary structure of the S4 helix could be important for the function of the protein by turning the arginine residues away from the lipid membrane as they start the motion towards the intracellular side in response to the hyperpolarization of the membrane. The easy solution would be if the side chains are simply pulled down by the hyperpolarizing field, essentially dragging the helix into 3_10_ helix. However, this would not explain why the transition *in vivo* can take up to milliseconds, or why there are separate open-active and open-inactive states. In the hyperpolarization simulations, the backbone and side chain rotation appears to be quite correlated and slow, and the 3_10_ helix is even growing from the intracellular side. As mentioned above, this rather points to an entropic effect of packing S4 to the remaining VSD helices, somewhat akin to finding a keyhole in the dark—it takes time, and pushing harder will not help. It also supports an overall ‘screwing’ motion of S4, but in particular a model where S4 first might have to transition to 3_10_ helix (AL to A) for the actual closing motion (A to R) to be possible, as recently also suggested by Villalba-Galea et al. [27].

### Protein-Membrane Interactions

**Figure 8:**
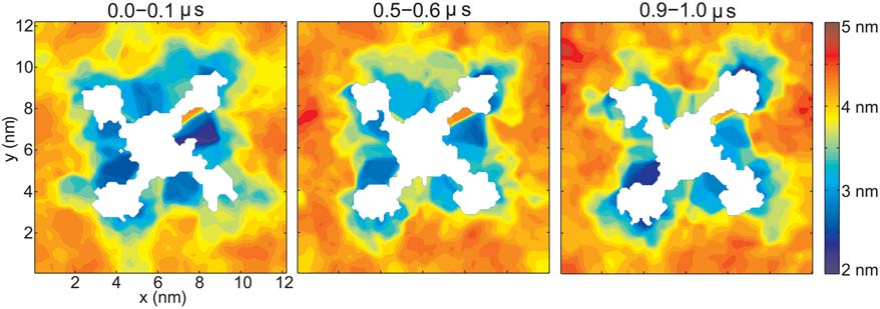
Lipid bilayer thickness. Distance between sn2 carbons (second carbon of the glycerol backbone) on opposite sides of the bilayer, calculated on a grid in the xy-plane (viewed from the intracellular side) and averaged over 100 ns in the beginning, in the middle and in the end of the simulation. White areas are representing grid-points where the lipid densities are low in both leaflets, i.e., protein regions.

The lipid bilayer thickness was studied by calculating time-averaged bilayer thickness between sn-2 carbons in the glycerol group (the carbon at the branching point between the two acyl chains and the head group) on a grid in the plane of the membrane (the xy-plane). The result is shown in Figure 8. Membrane proteins have been shown to induce large distortions in the surrounding bilayer in some cases and that the lipid membrane can modulate protein function [61]. Clearly, the transmembrane protein has a large effect on the bilayer thickness; in general, the thickness is obviously reduced close to the protein and in particular in the grooves between neighboring VSDs (down to ∼25 Å) compared to the bulk thickness (40–50 Å).

**Figure 9:**
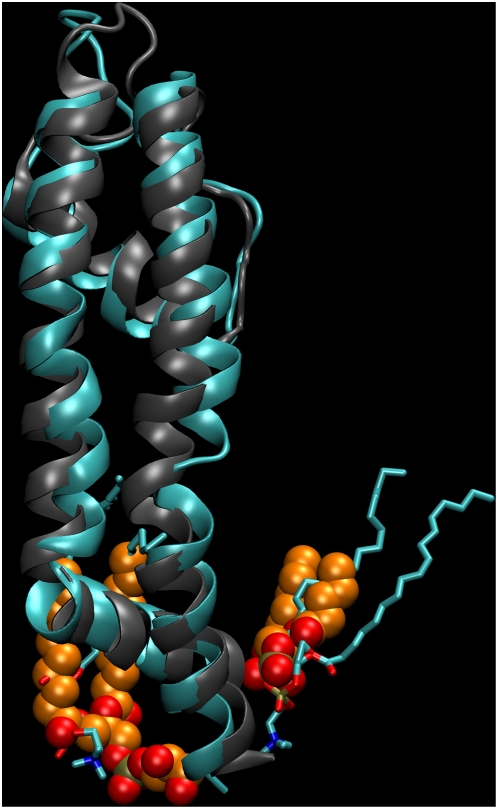
Comparison of lipid structure between a simulation snapshot and the new Kv1.2 chimera crystal structure. A sideview of the S4–S5 linker to S6 region of the crystal structure (gray) superpositioned on a snapshot of the simulation (cyan). Lipid molecules of the crystal structure are shown as van der Waals spheres. Two lipids molecules from the simulation at corresponding positions are shown in stick representation.

The Kv1.2 chimera structure [7] has highly resolved lipid molecules in the crystal forming a bilayer-like structure, especially in the grooves between laterally protruding neighboring VSDs, indicating that these lipids are an integral part of the protein structure and probably contribute to its stability. This suggest strong lipid-protein interactions in those regions which in turn support the data that shows that these parts of the lipid bilayer are very important for lipid and lipid-soluble channel-regulators, such as drugs and toxins [62]–[64]. Even though the number of resolved lipid molecules is rather low in the crystal structure, a direct comparison to the simulations is tempting. In addition to several lipid acyl chain fragments, three polar lipid head groups are resolved in the unit cell (containing a single subunit); one in contact with the C-terminal part of S6, the second wedged between the VSD and the S4–S5 linker, both part of the intracellular leaflet, whereas the last is located close to the P-loop on the extracellular side. This latter region had already been proposed to be a lipid-interaction site due to lipid head group electron density in the KcsA crystal structure [65]. The lipid close to the S4–S5 linker in the intracellular leaflet was noted to have a considerable different level of burial in the bilayer. By mapping snapshots of the simulation onto the crystal structure we estimate a thinning *specifically on the intracellular side* of at least 8 Å in this region. These two lipid molecules are shown in Figure 9 (lipid close to the S4–S5 linker on the right), together with two POPC lipids from the simulation at very similar positions—note that this packing was not assigned in the starting conformation. The simulation suggests a thinning, not only of the same magnitude, but also in the same region of the bilayer.

The lipid-exposed positively charged arginine residues in S4 perturbed the membrane bilayer in their neighborhood because of their hydrogen-bonding to lipid head groups. However, they *never* interacted directly with the hydrophobic phase. On average, lipids were dragged down to be able to form these interactions and the bilayer hence became thinner in this region which can be seen by the mostly blue-colored regions of the membrane in Figure 8, close to the PD at the right edges of the VSDs as viewed radially from the protein pore (region at x,y-coordinates ∼(4,4.5) nm in plot, for subunit A). Such interactions between the positively charged arginine residues of S4 and the negatively charged lipid phosphodiester groups are crucial for the protein function, as they are stabilizing the open conformation [66]. Moreover, this significant thinning of the bilayer in the proximity of the voltage-sensing arginine residues further helps the electric field focusing [25],[67].

Other simulations have also reported on the thinning of the membrane with immersed voltage-gated ion channels or VSDs. In simulations of the VSD in POPC/POPG bilayer mixtures at 3∶1 molecular ratio, Sands et al. found a thinning of the bilayer of ∼7 Å close to the voltage sensor compared to bulk (measuring the P-P distance of opposing phospholipids) [38]. In simulations of an isolated S4 helix (from KvAP) in a POPC bilayer, Freites et al. [68] found such a deformation of the membrane in the vicinity of the peptide where the hydrophobic core was reduced to a mere 10 Å. In particular, they noted that one lipid in a simulation snapshot spanned the entire membrane in a configuration resembling that of a monolayer. Roux and coworkers [26] also reported significant membrane thinning at the intracellular side in their simulation of Kv1.2 due to increased interactions between basic residues and (DPPC) lipid head groups. Although these studies are hard to compare due to differences in methodology, lipid and protein compositions, thinning of the bilayer around the VSD is present in all cases and the phenomenon most likely has functional importance because it both stabilizes the open state and focuses the electric field around the voltage-sensing arginine residues of the S4 helix.

**Figure 10:**
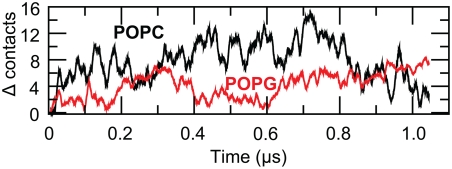
Change in number of contacts between the protein and lipids. A contact is defined between the protein and a lipid if at least one protein atom is located within 9 Å from the phosphorous atom in the lipid head group. The absolute initial number of contacts are 102 and 39 for protein-POPC and protein-POPG, respectively.

To characterize the nature of the lipid-protein interactions, the number of POPC and POPG lipids within 9 Å of the protein surface (measured from the phosphor atom in the lipid head group) was analyzed and compared between the first (random) and last (equilibrated) frames of the simulation, see Figure 10. The POPC/POPG ratio at the start of the simulation is 2.61 while it decreases to 2.28 at the end, compared to the overall ratio of 2.82 (313/111), clearly indicating an enrichment of negatively charged POPG lipids close to the protein. A closer look at the data reveals that the contribution to the POPG enrichment is mainly due to increased interactions between POPG and positively charged residues on the intracellular side of the protein, far away from the voltage-sensing arginine residues in the open state. This might not be critical for the protein function, but experimental studies have shown that the presence of the phosphodiester moiety of the lipid head group is important for the stabilization of the open state of the voltage-dependent K^+^ channels [66]. Rather, this enrichment might simply be explained by electrostatics; the charge distribution of the protein differs significantly between the intracellular and extracellular sides of the membrane with a clear overweight of positively charged residues at the intracellular side, which hence attracts negatively charged POPG lipid molecules. Biological relevance of specific interaction sites of anionic lipids can not be ruled out, however, since they have been detected in KcsA [69] and they are thought to modulate the selectivity filter stability [70]. In the same spirit, cardiolipin binding sites have been reported for a number of membrane proteins such as the ADP/ATP carrier, and a cardiolipin molecule has also been found in the structure of the cytochrome bc1 complex [71]. Finally, they could be important for accessibility of lipid-soluble protein functional regulators binding at or in close proximity to these sites.

## Discussion

The environment of the charged arginine residues in S4 after equilibration confirms previous reports that the two most extracellular arginine residues are only partly exposed to the lipid environment where they hydrogen bond to lipid head groups, in addition to transient interactions with water molecules in the extracellular VSD crevice and protein residues of the neighboring pore domain. The downstream two arginines were found to participate in stable salt-bridges with negatively charged residues from the neighboring S1 and S2 helices, forming a structural barrier separating the extracellular and intracellular water cavities within the voltage-sensing domains.

The initial 30–40° rotation of the upper part of S4 in all subunits (both with and without hyperpolarization applied) indicates that the equilibrium state in a bilayer is likely somewhat different from the crystal structures. The rotation partly moves the two arginine residues within this segment away from the membrane-exposed surface of the VSD, towards its interior. Recently, Lewis et al. [53] presented a study that supports the notion that the voltage sensor in the crystal structure might not have the exact orientation expected in the open-activated state, based on experimentally derived distance-constraints between the most N-terminal arginine in S4 (R294) and residues in the S5 helix of the pore domain, but they should be rotated by ∼37°. Our simulations agree virtually perfectly with this, and enabled interactions (although transient) between R294 and relevant S5 residues.

However, since this rotation occurs also in the simulation without external applied field (which should probably correspond to the AL state, if that is indeed the state of the crystal structure), this motion might not be representative of the AL to A transition, but rather an equilibration of the X-ray structure due to different environment (e.g., ion concentrations) in the crystal vs. the simulation. It is not trivial to say which one of these is closer to the native state. To focus on possible simulation shortcomings, one could, e.g., argue that the rotation might be be due to incomplete initial lipid packing around the protein, which is important for the stability of the S4 helices. While theoretically possible, we find this somewhat unlikely since the ion channel coordinates were restrained for the first 20 ns of simulation to pack lipids efficiently around the protein, and the rotation subsequently took another ∼50 ns to complete. Furthermore, the membrane used for solvation did not have the four-fold symmetry of the ion channel, so it is improbable that incomplete local solvation would cause quantitatively similar rotation in all four VSDs.

The slower, large-magnitude, rotation of S4 observed in the VSD of subunit C (roughly 120°) is quite striking, and most likely caused by the hyperpolarization of the membrane since no corresponding rotation is observed in the simulation without the applied electrical field. Equally important—this rotation is closely coupled to most of S4 forming 3_10_ helix (17 residues), which we hence can attribute to the hyperpolarization with fair confidence. Together, this has the effect that the positively charged residues in S4 align on the same phase of the helix, all pointing to the VSD interior. While some caution is advised considering the limited amount of data, this does look like an early stage of the voltage sensor transitioning. This is supported by considerable axial rotation of the S4 helix (as much as ∼180° in recent disulfide and metal bridge experiments [57]) and a change of the tilt angle of the helix [72].

A shorter stretch (two turns) of 3_10_ helix is also present in the new chimera Kv1.2/Kv2.1 structure [7], and the authors have suggested it could be important for gating. In a new experimental paper by Villalba-Galea et al. [27] this idea has taken further and they propose a mechanism of gating where the S4 helix totally adopts a 3_10_ conformation as it moves between the resting and activated states (R and A). Moreover, their data suggest that the slow inactivation of the protein (the A to AL transition) takes the S4 helix from the 3_10_-conformation to *α*-helical in the open-inactivated state. Interestingly, since the crystal structures of the Kv-channels are thought to be in the open-inactivated state, this seems to support the transition observed, which in that case would correspond to the backward direction from open-inactivated AL state towards the activated A state (S4 forming 3_10_ helix), which later would enable the A to R transition since a tighter helix with aligned charges could be easier to translate vertically.

Since stepwise activation of the VSDs has been observed experimentally [13],[73], we expect similar transitions to later occur in the remaining three subunits too. However, since even the transition motion takes close to half a microsecond to complete, even these fairly long simulations are at the very lower boundary of the timescales required to overcome the free energy barriers involved.

Since gating is a reversible process, the hyperpolarization should return the protein to the closed resting state. However, it is not obvious that this mechanism of charge transfer is strictly the reverse of activation, in particular not when starting from the open-inactivated state. It is for instance likely that the closing process too is initiated in the VSD, and conformational changes propagated to the channel such that the entrance to the ion-conducting pore closes. In this case, it is also quite possible that S4 first might have to rotate away from the lipids when destabilized by the hyperpolarization, and later translate down due to entropic packing (whether this corresponds exactly to the AL-A-R transition or not is a separate question). While high potential/temperature or coarse-grained models are extremely useful from an understanding point of view, they can also easily alter this order of events or produce artificial motion. In fact, based on recent experiments [27] it is doubtful that a VSD should be able to transition (in particular not directly from AL to R states) on 10–100 nanosecond timescales while not changing secondary structure!

So, which is the chicken and which is the egg? Does the 3_10_ helix formation drive the rotation or vice versa? Unfortunately this is not easy to resolve even from simulations since the two appear to be extremely coupled—the 3_10_ helix of subunit C extended from the C-terminal side of the helix with a duration directly correlated with the large rotation after hyperpolarization. The seed for this alteration in structure seems to have been a hydrogen bond forming abruptly between K306 in S4 and D259 in S3, putting a local strain on the S4 helical backbone which subsequently was mediated ‘upwards’ in the S4 helix. Prior to this, the VSD helices became less tightly packed transiently as reflected by a 10% increase in the radius of gyration around the z-axis, possibly pushing the structural flexibility needed to initiate the 3_10_ formation in this otherwise tightly packed central part of the VSD. On the other hand, the first part of the rotation at the N-terminal end of S4 began directly as the positional restraints were released and cannot be attributed to the change in secondary structure which started after about 0.2 µs in subunit C. Further, if the subsequent large rotation in subunit C was caused solely by the 3_10_ formation, then one would expect that the more C-terminally located R297 would start to rotate *before* R294. In practice, we believe it is a collective entropic process of re-packing S4 against the adjacent helices when the field changes, which would explain both the slow transition and why S4 can be transiently stable as 3_10_ helix in the open-active state.

The interactions between lipids and the membrane protein constitute an entire chapter by itself. Generally speaking, the coupling between membrane protein activation and membrane composition has been reported previously [74], illustrating the importance of specific lipid-protein interactions as well as the effects of membrane elastic properties on protein structure and dynamics. The thinning of the bilayer in the grooves between neighboring VSDs is evident, and this is actually also the region with resolved lipids in the new crystal structure of the chimera Kv1.2 protein [7], indicating favorable interactions with the protein. There are several experimental reports of functionally important interactions between the voltage sensor and lipids, both for stabilizing the open state of the voltage-dependent K^+^ channels [66] and for the focusing of the electric field around the voltage sensor [25],[67], that are mapped to this area of the bilayer. Additionally, interaction sites with protein function regulators, such as drugs and toxins, have been shown to be located on the protein surface facing these lipid regions [62]–[64]. Hence, the bilayer behavior within these grooves seems to be of a very different nature compared to the bulk lipid bilayer and other parts of the membrane close to the protein. Quantifying these differences more thoroughly is something we will focus on in a future project.

## Supporting Information

Figure S1 Average actual electric field and potential in the 1 microsecond simulation. Shown as a function of z-position with and without the applied field. The field and potential is calculated by single and double intergration, respectively, of the charge density according to the Poisson equation. Due to depolarization at the water/lipid interface, virtually the entire potential shift occurs over the membrane part of the system.
(0.71 MB TIF)

Figure S2 Charged residue dynamics in S4 for subunits B and D. Panels A&F show the relative Cα translation along the membrane normal, with positive direction towards the extracellular side. Note the difference in scales on the y-axis. Panels B/G&C/H indicate rotation of Cα and the outermost heavy atom around the local helix axis, respectively (clockwise rotation being positive when viewed from the N-terminal end of the S4 helix). Panels D/I&E/J display the number of hydrogen bonds formed with the rest of the protein and lipids, respectively.
(2.75 MB TIF)

Figure S3 Dynamics of charged amino acids in subunits A and C without external field (Compare Figures 4&S2). Panels A&F show the relative Cα translation along the membrane normal, panels B/G&C/H rotation of Cα and the outermost heavy atom around the local helix axis, and Panels D/I&E/J the number of hydrogen bonds formed with the rest of the protein and lipids. Interestingly, all subunits show limited rotation (30–40 degrees) even without applied field, indicating the membrane environment could be slightly different from the crystal structure.
(1.92 MB TIF)

Figure S4 The secondary structure of subunits B and D as calculated by DSSP. Note that no 3_10_ formation in S4 is present (compare Figure 7).
(2.97 MB TIF)

Figure S5 DSSP secondary structure of subunit A and C without external electric field. There is no significant growth of 3_10_ helix contents in S4.
(0.93 MB TIF)
