# Supplementary figures and images for: Conformational Changes and Slow Dynamics through Microsecond Polarized Atomistic Molecular Simulation of an Integral Kv1.2 Ion Channel
Source: PLoS Comput Biol. 2009 Feb 20;5(2):e1000289. doi: 10.1371/journal.pcbi.1000289 (PMC2632863; doi:10.1371/journal.pcbi.1000289)

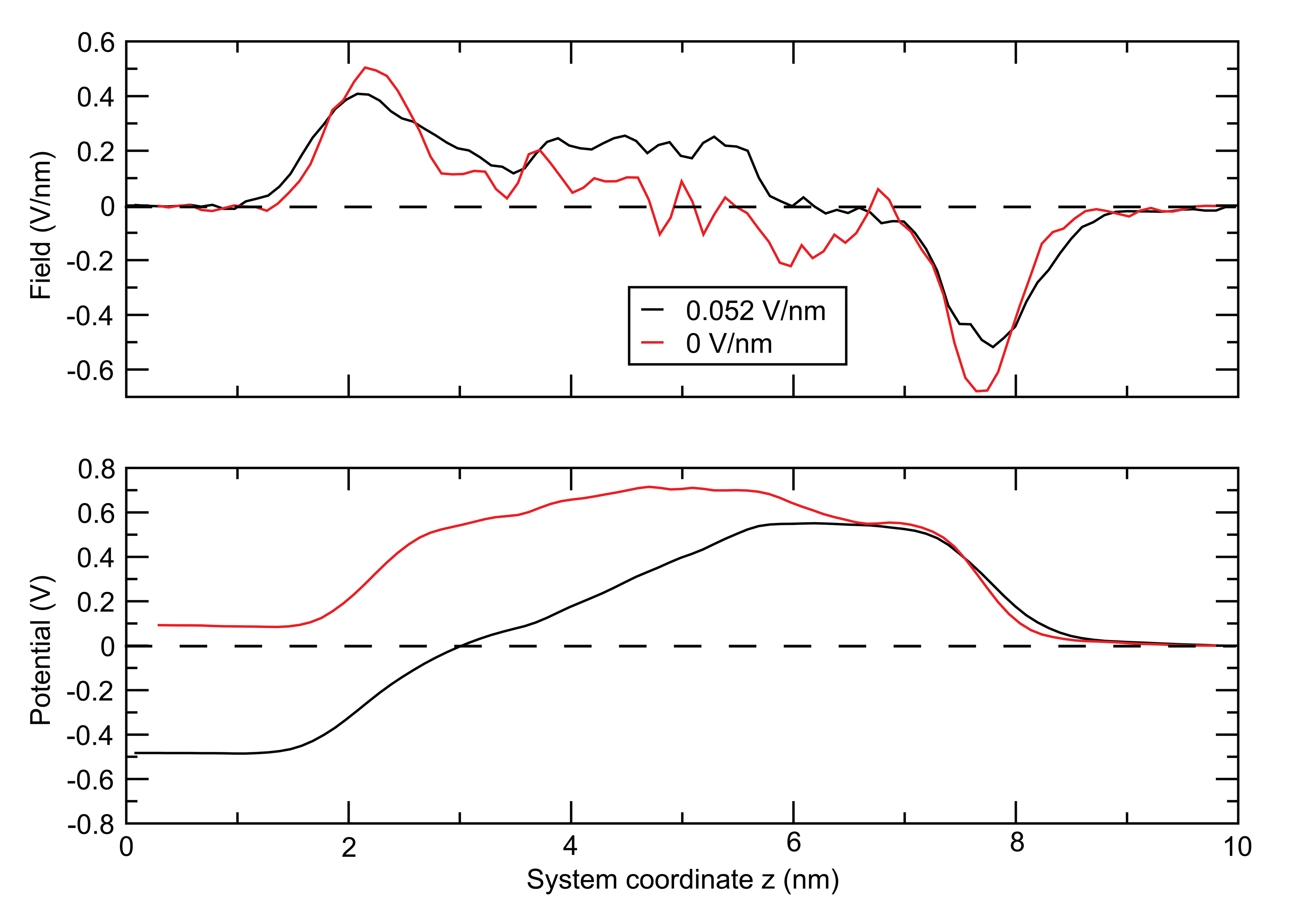

Supplement: Figure S1 — Average actual electric field and potential in the 1 microsecond simulation. Shown as a function of z-position with and without the applied field. The field and potential is calculated by single and double intergration, respectively, of the charge density according to the Poisson equation. Due to depolarization at the water/lipid interface, virtually the entire potential shift occurs over the membrane part of the system. (0.71 MB TIF) [file pcbi.1000289.s001.tif]

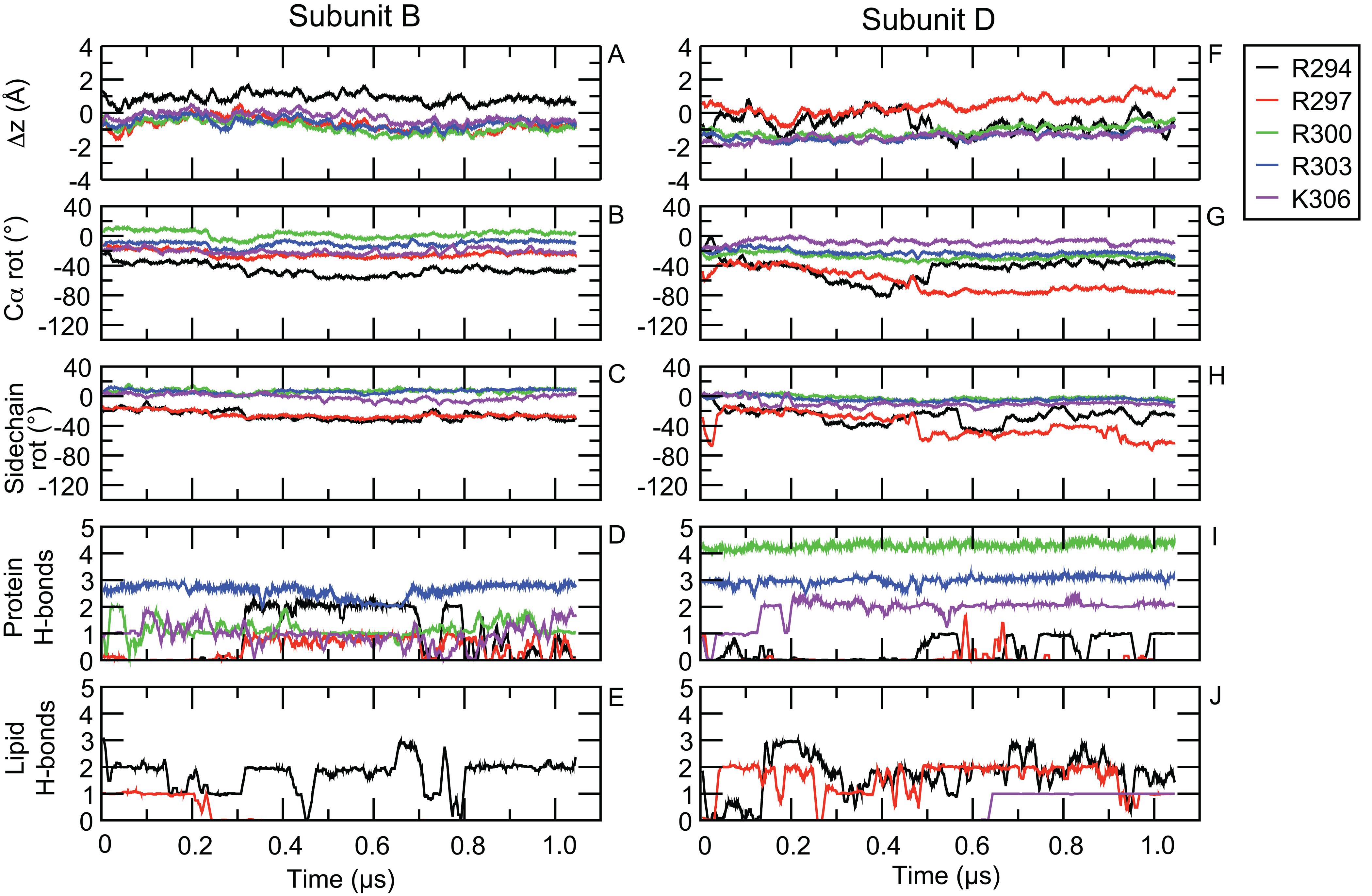

Supplement: Figure S2 — Charged residue dynamics in S4 for subunits B and D. Panels A&F show the relative Cα translation along the membrane normal, with positive direction towards the extracellular side. Note the difference in scales on the y-axis. Panels B/G&C/H indicate rotation of Cα and the outermost heavy atom around the local helix axis, respectively (clockwise rotation being positive when viewed from the N-terminal end of the S4 helix). Panels D/I&E/J display the number of hydrogen bonds formed with the rest of the protein and lipids, respectively. (2.75 MB TIF) [file pcbi.1000289.s002.tif]

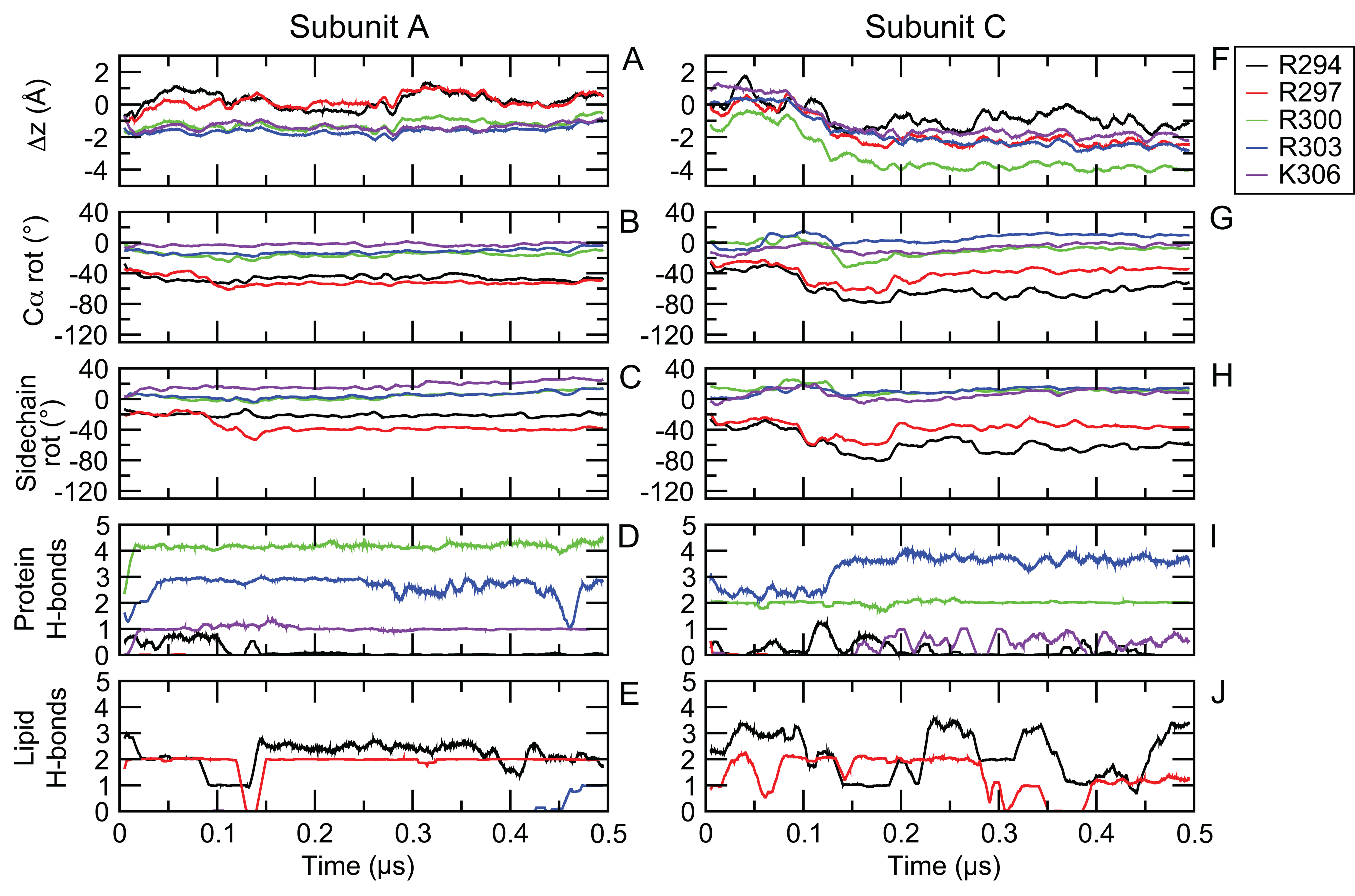

Supplement: Figure S3 — Dynamics of charged amino acids in subunits A and C without external field (Compare Figures 4&S2). Panels A&F show the relative Cα translation along the membrane normal, panels B/G&C/H rotation of Cα and the outermost heavy atom around the local helix axis, and Panels D/I&E/J the number of hydrogen bonds formed with the rest of the protein and lipids. Interestingly, all subunits show limited rotation (30–40 degrees) even without applied field, indicating the membrane environment could be slightly different from the crystal structure. (1.92 MB TIF) [file pcbi.1000289.s003.tif]

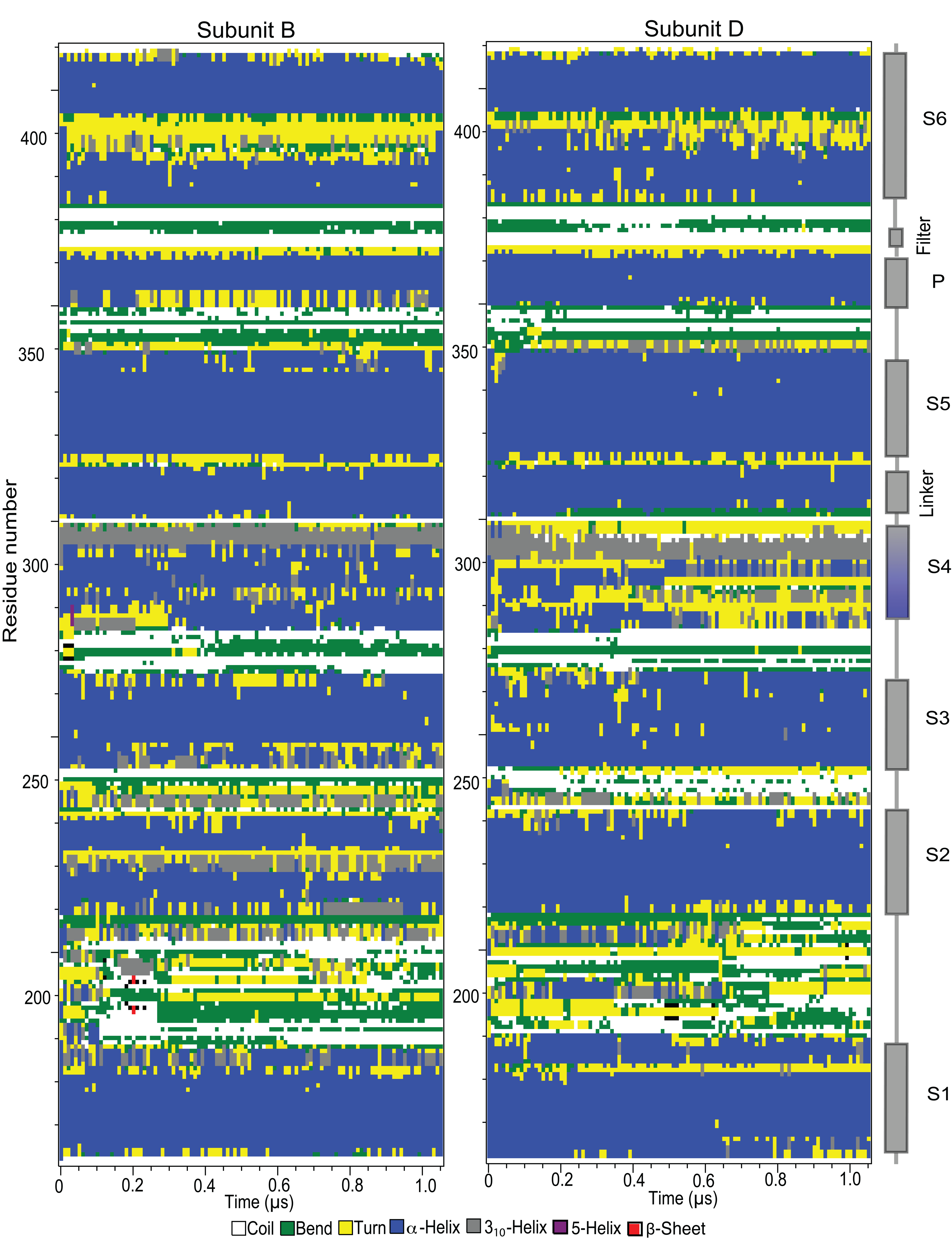

Supplement: Figure S4 — The secondary structure of subunits B and D as calculated by DSSP. Note that no 310 formation in S4 is present (compare Figure 7). (2.97 MB TIF) [file pcbi.1000289.s004.tif]

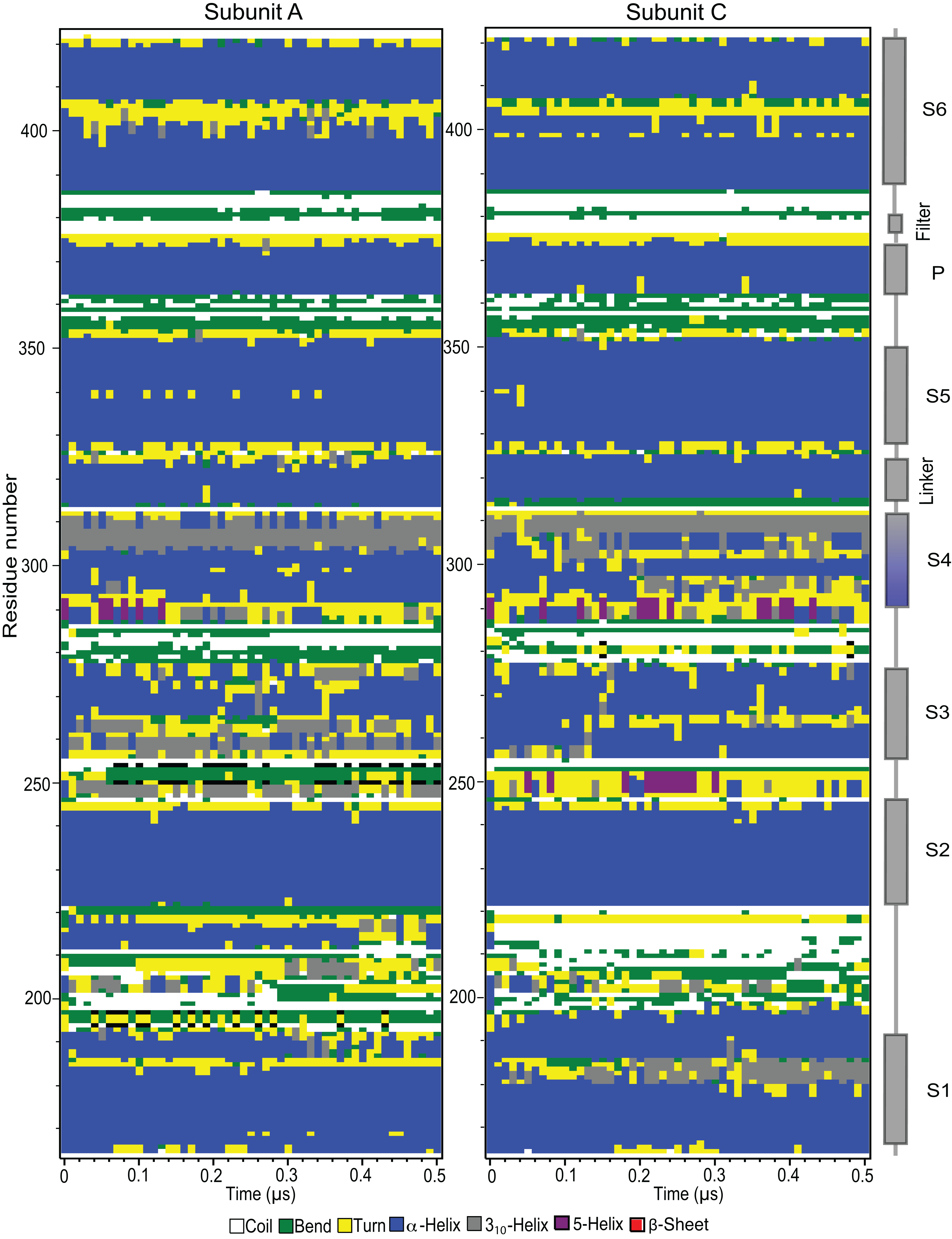

Supplement: Figure S5 — DSSP secondary structure of subunit A and C without external electric field. There is no significant growth of 310 helix contents in S4. (0.93 MB TIF) [file pcbi.1000289.s005.tif]
